# An Insight into the Current Understanding of Status Epilepticus: From Concept to Management

**DOI:** 10.1155/2021/9976754

**Published:** 2021-07-13

**Authors:** Khouloud Abdulrhman Al-Sofyani

**Affiliations:** Department of Pediatric, Pediatric Intensive Critical Care Unit, King Abdulaziz University, Jeddah, Saudi Arabia

## Abstract

Status epilepticus (SE), a subset of epilepsy, represents a debilitating neurological disorder often associated with alarming mortality and morbidity numbers. Even though SE is one of the extensively researched topics with conspicuous data available in the literature, a scientific gap exists in understanding the heterogeneous facets of the disorder like occurrence, definition, classification, causes, molecular mechanisms, etc., thereby providing a defined management program. Cognizance of this heterogeneity and scientific limitation with its subsequent correlation to the recent advancements in medical and scientific domains would serve not only in bridging the gap but also in developing holistic and prompt management programs. Keeping this as an objective, an extensive literature survey was performed during this study, and key findings have been shared. The present study provides a semantic and perspective synopsis toward acknowledging the diversified nature of SE and its variants with respect to their definition, classification, etiology, diagnosis, and management.

## 1. Introduction

Epilepsy is a neurological disorder marked by sudden recurrent episodes of sensory/motor disturbances, impaired consciousness due to abnormal electrical activity in the brain. Status epilepticus (SE) is a medical condition where the seizures are prolonged and require urgent medical intervention, resulting in long-lasting consequences and fatalities if left untreated or unattended. Approximately 1.3–16% of the subjects who suffer from epilepsy eventually deteriorate to status epilepticus (SE) condition [1].

Status epilepticus is one of the most prevalent pediatric neurological emergencies, only second to stroke, resulting in neurological intensive care unit (ICU) admission. Global statistical data reveals the incidences of SE are around 50 patients per 100,000 population per year with a mortality rate of around 2.5% [2]. The exact meaning of status epilepticus (SE) is context-specific. However, SE can be simply phrased as prolonged seizures demanding urgent medical intervention, which may result in higher morbidity and mortality rates if either left unattended or delay in the course of action [3].

Although SE could happen at any age, its impact is most pronounced in the vulnerable age groups comprising children and older adults [4–7]. SE is predominantly prevalent in neonates and infants as compared to the elderly adult population [8]. Incidence of SE in children below one year of age was found to be almost 150 per 100,000 population, while in the age group 1–5 years, the incidence rates declined to <25 per 100,000 people before rising to >50 per 100,000 for people above 40 years of age [5]. In another survey, the prevalence of SE in children aged <5 years was 7.5 per 100,000 and 22.3 per 100,000 for the elderly population [5]. Continuous EEG surveillance is one of the significant reasons for the observed higher prevalence rate of SE in children [4, 9].

Concerning the socioeconomic parameters, higher SE prevalence is observed in developing countries as compared to the developed countries. The higher prevalence of SE in developing countries is due to scarcity of proper health care infrastructure, sustainable management policies, compromised hygienic conditions, and inadequate resources, which is also reflected in the higher mortality index. The available data suggests that almost 50 million people get affected with SE, of which 80% reside in developing countries [10–13].

For a long time, SE has been a subject of research, and significant literature and data available in this domain have helped researchers understand this disorder concerning its types, causes, symptoms, management, etc. The current article attempts to provide a holistic understanding of the SE and its variants, keeping abreast with the current understanding and practices observed in the scientific and medical domains.

## 2. Definition

Contemporary with the then medical knowledge, the definition of SE has been evolving since 1981 to date. It is considered that both the long intervention time (around 60 min) and the brief intervention time (around 5 min) are equally effective during the management of SE and its variants. Taking awareness of these two time points and different SE types, the International League Against Epilepsy Task Force for Classification of SE (ILAE) has proposed a revised definition [14]. As per the newly revised definition, the two time point indicators for SE are T1 and T2. T1 is the time point during which the treatment should be initiated. The duration of T1 is 5 min for the convulsive type of SE, where there is a high probability for prolonged seizures beyond the 5 min timeframe (Table 1). T2 is the time point after which the long-term consequences of seizures may occur, and the duration of T2 continues to be 30 min (Table 1) [5]. According to the ILAE model, it can be concluded that the urgency of management time or intervention time is primarily dependent upon the type of SE (Table 1). This definition takes knowledge of the importance of time variability in treating status epilepticus regarding the seizure type [5, 14, 15].

Considering this analogy, it is imperative to further discuss SE and its types.

## 3. Classification

Classification of complex neurological disorders like SE should ideally be based on the underlying neurobiology, leading to natural classes or entities. A classification of SE primarily serves four purposes:

1. To facilitate the development of common terminologies enabling proper and effective communication among the clinicians
2. To improve patient's treatment on the basis of the current knowledge of pathophysiology, prognosis, etiology, and age
3. To conduct epidemiologic studies to understand the consequences and prevention of SE
4. To promote basic research in identifying natural classes of SE, which in turn will lead to true scientific classification; it is also worth understanding that the present classification is reflective of our current knowledge on the subject, SE

SE has been classified based upon different parameters like semiology, duration of the epileptic episode, etiology, clinical manifestations, etc. Future advances in basic, epidemiologic, and clinical research will most likely result in modifications and major revisions in the existing classification [14].

A consolidated perspective of SE classification based on the literature survey is summarized in Table 2.

Relevant to the paper's current context, clinical classification of SE is further discussed herein, which would provide the readers an edge in understanding the chronological description of the subsequent sections.

### 3.1. Generalized Convulsive Status Epilepticus (GCSE)

Generalized convulsive status epilepticus (GCSE) is the most common type of SE that results in higher mortality and morbidity, accompanying extremities in rhythmic jerking. Diagnosis of GCSE is based upon loss in the consciousness of the subjects accompanied by convulsive movements. Retained consciousness among the subjects distinguishes convulsive SE (like simple partial or focal motor status) from asymmetric secondary generalized SE. Characteristic observable traits associated with GCSE include the following:

  Generalized tonic-clonic movements of the extremities
  Mental status impairment (coma, lethargy, confusion)
  Focal neurological deficits in the postictal period (e.g., Todd's paralysis)

More than half of the SE patients respond to treatment with a single antiseizure medication. The other half of the patients represent an ongoing medical emergency, where two or more antiepileptic drugs are required in managing those patients. Experimental data indicate that failing to treat GCSE aggressively during the initial period may increase the likelihood of the patient being unresponsive to one or two medications [18, 19].

### 3.2. Nonconvulsive Status Epilepticus (NCSE)

NCSE can also be termed “subtle status epilepticus.” NCSE typically follows uncontrolled GCSE and is often reported in critically ill patients admitted in the ICU [10]. Continuous EEG monitoring is of utmost importance to diagnose it.

An array of symptoms with varied patterns are observed in these subjects, including delusions, delirium, agitation/aggression, facial twitching, automatisms, blinking, nausea/vomiting, crying, echolalia, laughter, nystagmus/eye deviation, perseveration, tremulousness, and psychosis. However, two distinct phenotypical attributes of NCSE have been described previously:

  The ‘‘wandering confused” patient presenting to the emergency department with a relatively good prognosis or chronic epileptic syndromes
  The acutely ill patient with severely impaired mental status, with or without subtle motor movements (e.g., rhythmic muscle twitches or tonic eye deviation that often occurs in the setting of acute brain injury)

Negative symptoms observed in the NCSE cases include anorexia, aphasia/mutism, amnesia, catatonia, coma, confusion, lethargy, and staring. Most NCSE cases are treated with low and recurrent doses of benzodiazepines, phenobarbital, and phenytoin [10, 19–21].

### 3.3. Refractory Status Epilepticus (RSE)

Differences exist among the scientist and medical practitioner's fraternity regarding the most appropriate definition of RSE. Although not unanimously accepted, the most common definitions of RSE observed in the literature are as follows:

  RSE is defined as SE which is refractory to intravenous antiseizure medications, essentially a benzodiazepine, or
  It could also be referred to the cases where patients commonly do not respond to standard treatment regimens for SE as directed by the guidelines [22], or RSE has also been defined based on the duration of seizure that could last for around 1 or 2 hours [10]

Around 23–43% of subjects predisposed to SE develop RSE. RSE is characterized by severe impairment of consciousness, leading to short-term mortality but high morbidity. Underlying etiologies attributing to RSE are acute and severe encephalitis, a massive stroke, or rapidly progressive primary brain tumor. Phenobarbital, midazolam, and propofol are the pharmacological agents that are usually used in RSE management [10, 22].

### 3.4. Superrefractory Status Epilepticus (SRSE)

This term was coined for the first time at the “Third London-Innsbruck Colloquium on Status Epilepticus” held at Oxford in 2011. SRSE is defined as SE that prolongs for 24 hours or more after the application of the anesthetics [10]. The above definition also includes those cases in which SE recurs after weaning of the effects of anesthetic agents. High mortality and morbidity in the patients are key features of this uncommon clinical problem. The actual prevalence of SRSE is unknown because of a paucity of data. Delaj et al. (2017) reported a 4% incidence of SRSE in the study conducted among 804 subjects, while the other data reported in the literature confirms the incidence of around 12.2–22% of SRSE among all SE episodes [23]. In a study in India, encephalitis was reported to be a key determinant cause for the progression of SE to SRSE [10].

### 3.5. New-Onset Refractory Status Epilepticus (NORSE)

NORSE is another rare SE condition, which is characterized by a prolonged period of refractory seizures with no readily identifiable cause in otherwise healthy individuals. Initially, the absence of a proven etiology was considered mandatory for NORSE diagnosis, although no such conclusive study could affirm this finding. Recent reports have suggested autoimmune encephalitis (paraneoplastic or nonparaneoplastic) or unidentified viral infections could be the common causative factors for NORSE. Although the outcome is generally poor for the NORSE patients, the outcome improves after follow-up; however, epilepsy develops in most of the patients [10, 16, 24].

### 3.6. Febrile Infection-Related Epilepsy Syndrome (FIRES)

Many researchers consider FIRES as a subcategory of NORSE. It is a fatal epileptic syndrome prevalent among children aged between 3 and 15 years. The subjects suffering from FIRES experience a nonspecific febrile illness followed by the onset of seizure activity lasting for around 1–2 weeks. Currently, the treatment of FIRES is not well established as most of the patients are unresponsive to the current medications. The role of immunomodulatory therapy is also controversial. In some subjects, the ketogenic diet has shown limited benefits [24, 25].

A consolidated summary of SE and its type along with the details of their diagnosis and management are presented in Table 3 [10].

## 4. Causes/Etiology

Before discussing the several factors responsible for the seizures gradually culminating into SE, it is imperative to briefly understand inhibitory and stimulatory neuronal pathways operating at the cellular levels. Primarily, failure of a seizure to stop is due to the imbalance between the inhibitory GABA (gamma-aminobutyric acid) pathway and the excitatory glutamate-mediated pathway. The inhibitory mechanisms are either temporarily diminished/sensitized or permanently damaged during SE, resulting in a prolonged period of epileptic bursting [22].

Receptors exist in a highly dynamic state at the cellular level facilitating their movement along the axonal membrane. Receptor trafficking via internalization of the surface-positioned GABA receptors with a concomitant increase in the number of glutamatergic receptors at the cell surface results in persistent excitatory pathways with decreased or nonoperational limiting pathways. Thus, in general, any pathological condition which triggers an acute symptomatic seizure can cause an SE. Examples of such pathological conditions are neurological and systemic infections, acute vascular events, traumatic brain injury, and immune, metabolic, or toxic encephalopathies [10, 28].

Significant animal data pertaining to the pathogenesis of SE is available in the scientific domain. The causes of SE are numerous. In general, etiologies are divided into

  Cryptogenic/unknown: when a clear cause is not known
  Remote symptomatic: when it is secondary to a prior insult such as stroke or head trauma
  Acute symptomatic: when there is an acute neurological or systemic or progressive neurological disorder

The etiology of SE varies with age of the subjects. In adults, the most common causes of SE include trauma, tumor, vascular disease, alcohol withdrawal, and noncompliance with antiseizure medications whereas, in the pediatric population, the most common causes are unknown and remote symptomatic causes [4, 6, 7].

### 4.1. Continuous EEG Surveillance

Approximately 10–40% of seriously ill children who experience continuous EEG (cEEg) surveillance suffer from electrographic seizures, identified through single and multicenter inquiry. In addition, approximately one-third of children with significant electrographic seizure load are identified as epileptic. A systematic review of 550 chronically ill children in 11 tertiary care hospitals in the United States and Canada, who underwent EEG surveillance, was the first continuous EEG monitoring study at the pediatric intensive care unit. In 30 percent of the tracked infants, electrographic discharges occurred. Among those with electrographic seizures, 33% of the infants had an EPS that was described either as more than 30-minute seizures or as short repeated seizures for a total of 30 minutes in a period of one hour. In that study, one-third of the children with electrographic seizures had just EEG showing abnormalities with no clinical evidence of seizures [8, 9].

### 4.2. Infectious Factors

A greater prevalence of CNS infections and infestations leads to a higher SE incidence in developing countries than developed countries. Prevalence of all microbial infections, including viruses, bacteria, fungi, etc., has been observed throughout the literature [28].

In an earlier study, around 12% of the adult population predisposed to herpes simplex virus (HSV) infection or Japanese encephalitis was diagnosed with SE [29]. Comparatively, children suffering from encephalitis are more prone to develop SE [28].

A retrospective review of case records of patients with SE due to neurocysticercosis, during a period of 18 years, found that, in 38 (93%) of patients, convulsive SE was the presenting feature. The authors concluded that convulsive SE is rare even in countries endemic to neurocysticercosis [30]. The respiratory syncytial virus has occasionally been identified as the causative agent of SE in infants with bronchiolitis [31].

Pediatric SE has also been reported to be caused by a flu-like illness termed Q fever, which in turn is caused by *Coxiella burnetii*. Additionally, the DNA of *Chlamydophila psittaci* (Chlamydia) was also detected in the cerebrospinal fluid of an adult patient suffering from SE. Other neurological infections like bacterial meningoencephalitis, cerebral paragonimiasis, cerebral malaria, and dengue could also lead to SE although, currently, sufficient data are not present to prove this analogy [10, 28, 31].

### 4.3. Autoimmune Disorders

In children and adolescents, a leading autoimmune disorder is an anti-NMDA-receptor encephalitis, which presents nonspecific clinical manifestations such as fever or headache in the prodromal stage. In children, typically, the cerebral cortex is the affected region, while, in adults, inflammation has been observed to be localized in the temporal lobes. Limbic encephalitis is also being increasingly recognized in children and may often not occur as a paraneoplastic syndrome [28].

### 4.4. Genetic Factors

Prior studies have supported genetic predisposition as a cause of prolonged seizures [32, 33]. Dravet syndrome is a classic example of a genetic predisposition to SE, resulting from a mutation in the SCN1A gene that encodes for the subunit of voltage-gated sodium channels. Other genetic anomalies listed in this contest are sodium channelopathies, ring chromosome 20, pathogenic variants in polymerase-G or amino-acyl-tRNA synthetase genes affecting mitochondrial function, and Angelman syndrome [28, 34, 35].

Convulsive SE can result from several chromosomal aberrations, especially the Wolf-Hirschhorn (4p-) syndrome, ring chromosome 14, ring chromosome 20, the monosomy 1p36, and inversion-duplication of chromosome 15. However, there is no evidence of SE being the presenting symptom leading to the detection of these chromosomal aberrations. Down syndrome and Angelman syndrome, which are the result of mutations in the mitochondrial polymerase *γ* gene or genetic abnormalities in *β*-ureidopropionase, are usually diagnosed before the onset of convulsive and febrile SE [31].

### 4.5. Metabolic

Pyridoxine dependency, a defect in pyridoxine metabolism, may produce severe seizures. It is a disorder that is generally observed during the first hours of life, but intrauterine seizures and onset after the neonatal period have been observed too. Associated seizures are multifocal, either tonic-clonic or myoclonic. Mitochondrial myopathy encephalopathy, lactic acidosis, and stroke-like episodes (MELAS) may look like SE in patients without previous history of epilepsy. Most reported cases, though rare, belong to adult patients [31]. A 16-year-old girl and a 17-year-old boy with Wilson disease also displayed SE as the presenting symptom [36, 37]. Furthermore, in about 9% of the acute adrenoleukodystrophy cases, 1% of the cases present SE as a symptom [31].

### 4.6. Drugs and Toxins

Tricyclic antidepressant overdose, alcohol abuse, carbon monoxide intoxication, acepromazine, antihistamines (histamine H1 receptors antagonists), isoniazid, and organophosphates can elicit the seizure response. However, no particular substance is specifically associated with SE, although overdose of certain drugs, as mentioned above, is frequently associated with seizures. Occasionally, therapeutic doses of some commonly used drugs can provoke SE. In 2008, convulsive SE was reported in a 9-year-old girl with congenital hemiplegia who presented symptoms of convulsive SE shortly after beginning clonidine therapy for attention deficit hyperactivity disorder [31]. SE can also occur due to overdose of certain compounds. Cocaine and NDMA (3, 4-methylenedioxymethamphetamine, ‘‘ecstasy”) have occasionally been associated with SE in toddlers, especially in the presence of hyperpyrexia secondary to intoxication. Poisonous agents known to manifest as convulsive SE include fungicide maneb (manganese ethylene-bis-dithiocarbamate), camphor (an antiflatulence substance), neem oil (margosa oil), a traditional component of traditional medications in India, and tetramine, a banned neurotoxic [31].

## 5. Diagnosis

Guidelines are available on SE management for the use of antiseizure drugs (ASDs); however, no such uniform directives or guidelines exist for diagnosing SE [16]. To determine the underlying or prevailing pathology of SE, the Neurocritical Treatment Society Guidelines have prescribed several tests that should be executed immediately or done in parallel with the management [18]. The most common protocol followed during the diagnosis of SE could be categorized into a three-tier system [3].

1. Initial tests: these tests are the first tests to be performed as soon as possible, without any delay. These tests are important for the clinicians in understanding the pathology of SE and in deciding the probable first line of drug treatment to be initiated. The tests include a Fingerstick test, monitoring of vital signs, CT scan, and certain other tests like blood count, metabolic tests, AED levels, etc.
2. After initial analysis, certain diagnostic tests need to be performed subjectively upon the consultation and guidance of a physician. Such tests typically include MRI scan, lumbar puncture, comprehensive toxin analysis (e.g., isoniazid, antidepressants, theophylline, cocaine, organophosphates, cyclosporines, etc.), and other laboratory tests like liver function test, serial troponins, type and hold coagulation studies, and arterial blood gas levels
3. In certain variants of SE, where the exact etiology remains unclear and inconclusive upon the above two tests, the physician then recommends tests like antibody test and PCR test to understand the cryptogenic factor for the SE [31, 38, 39]

Out of all the diagnostic methods reported, continuous EEG tracking is widely practiced along with routine EEG monitoring under a physician's guidance. Timely EEG tracking is necessary to allow a clinician to take prompt action. American Clinical Neurophysiology Society suggests written EEG tracking plans and EEG monitoring reports at least twice a day for treating chronically sick adults and children during RSE, SRSE, and NORSE [3, 18, 40, 41]. According to the International League against Epilepsy Subcommittee, approximately 50% of person imaging studies have provided valuable data on the etiology of new seizures [41]. However, once the cause of the SE is specifically determined, rapid neuroimaging can be overlooked as it may elicit certain unwanted clinical implications among the patients. One study reported that 20% of brain CTs and 58% of brain MRIs were abnormal, and in 24% of the cases, change was observed in the neuroimaging of the patients undergoing acute management [39].

In certain variants of SE, it is imperative for the physician to opt for neuroimaging techniques over EEG-based diagnosis. For instance, neuroimaging (CT scans and MRI scans) should be undertaken more urgently in cases such as trauma, tumors, or cryptogenic SE [39].

Infections of the central nervous system are a common cause of childhood epileptic illness, responsible for 1–12% of subjects in developing countries [18]. Typically, these infections are acute or subacute bacterial meningitis, acute influenza meningitis, or encephalitis. For such reasons, initial evaluation involving lumbar puncture with cell count, gram staining, culture protein, glucose, and herpes simplex virus titer estimation by PCR should be undertaken [3]. Guidelines from the Infectious Disease Society of America recommend that the immunocompromised adults presenting with space-occupied lesion or shunt, papilledema, or focal neurological deficits should undergo head imaging before proceeding to lumbar puncture [3].

Autoimmune and paraneoplastic etiology account for 2.5% of total epileptic illnesses [42]. Regardless of the age, patients developing rapidly progressing symptoms accompanied by seizures or SE, including behavioral changes and memory deficits, should undergo serum and CSF analysis for antibodies, determination of IgG synthesis rate, intrathecal antibody synthesis, and IgG index along with traditional EEG-based monitoring [43].

The Neurocritical Care Society Guideline does not define genetic tests and usually does not play a part in the acute handling of the SE. However, several hereditary causes of epilepsy are associated with persistent SE, and others may have new SE without any particular neuroimaging or EEG diagnostic function [3]. Dravet syndrome is an inherited epilepsy associated with recurring epileptic conditions, particularly following vaccination, febrile status, and warm weather exposure. Besides Dravet syndrome, there are other epileptic disorders with contiguous gene aberrations. Next-generation sequencing methodologies can further commercialize gene-based diagnosis. Although genetic testing is not helpful in the early treatment of an epileptic disease, a genetic scrutiny should be undertaken by the physicians after a systematic and thorough assessment of epilepsy or SE of the patients where other etiologies have not been established [3, 34, 35].

## 6. Management

### 6.1. Pharmacological Perspective of ASDs

Antiseizure drugs (ASDs) constitute a mainstay in the treatment of SE. ASDs modulate the cellular pathways in CNS, thereby eliciting a pharmacological response. Therefore, understanding these cellular pathways in CNS is essential for gaining insights into the therapeutic effects of ASDs.

Epileptic episodes occur because of the imbalance between excitatory and inhibitory pathways in the CNS and they are briefly described as follows:

  Excitation of synaptic transport occurs during hyperpolarization of action potential effected by glutamate coupled to AMPA (*α*-amino-3-hydroxy-5-methyl 4- isoxazoleproionic), NMDA (N-methyl-D-aspartate) receptors, ionotropic Na^+^, and Ca^++^ channels
  Inhibition of synaptic transport occurs during depolarization of membrane potential and is effected by GABA (*γ*-aminobutyric acid) coupled to GABA-A receptors

Thus, drugs used during the management of SE essentially act by either enhancing the GABA-mediated pathway and/or inhibiting the glutamate and Na^+^/Ca^++^-mediated neuronal excitation.  Table 4 summarizes and categorizes different antiseizure drugs and their respective mechanisms [26].

### 6.2. Drugs Used in the Management of SE and Its Types

#### 6.2.1. SE

The neurocritical care community identifies benzodiazepines as an established and initial first line of treatment for SE. Intravenous administration of benzodiazepine is recommended, if possible. Benzodiazepines are also available in other presentations like buccal, intranasal, intramuscular, and rectal. According to recommendations from the American Epilepsy Society, intravenous lorazepam and rectal diazepam are also successful in preventing seizures lasting at least five minutes [2, 11].

The three drugs, and their respective doses, that have been used as first-line agents for aborting SE are midazolam (IM 10 mg/kg/>40 kg; 5 mg/13–40 Kg; single dose), lorazepam (IV 0.1 mg/kg/dose; repeat once as necessary), and diazepam (IV 0.15–0.2 mg/kg/dose; repeat once if required) [11]. A double-blind, randomized clinical trial, designed to compare lorazepam versus diazepam for treating pediatric SE showed that cessation of SE was observed in 72% of patients in the diazepam group and 73% in the lorazepam group until 10 minutes without recurrence within 30 minutes. In 16% of the patients in the diazepam group and 18% of the patients in the lorazepam group, aided ventilation was required. The authors also found that lorazepam patients were more prone to be sedated than their counterparts (67 percent vs. 50 percent). The analysis also showed no concrete evidence to suggest that lorazepam has a preferential effect compared to diazepam in that population [44].

Benzodiazepine administration results in respiratory depression and hypotension, and therefore, its use should be controlled and stabilized. The American Epilepsy Society guideline concluded that the most frequent adverse effect of antipseudomyopathy (level A evidence) was respiratory depression. No variation in respiratory depression was found in any administration route (level B evidence) among midazolam, lorazepam, and diazepam [11]. In 16% of diazepam and 18% of lorazepam treated subjects, a new randomized clinical review on extreme pediatric epilepsy confirmed that assisted ventilation was necessary. If 5–10 minutes after initial administration of benzodiazepine epileptic episode does not subside, the second dose of benzodiazepine should be given. However, it is also necessary to determine whether benzodiazepine has been administered before hospitalization because the respiratory suppression risk is enhanced due to overdosing of benzodiazepine [3].

The recommendations of the Neurocritical Treatment Society [18] and the American Epilepsy Society state the second step of therapy as “urgent,” and it contains second-line drugs [11]. Benzodiazepines alone can control the seizures in less than half of children if SE is already established [45]. Therefore, the Neurocritical Treatment Community recommends that another “emergency control medicine” benzodiazepine be administered to the subjects [18]. There is minimal evidence on the comparative effectiveness of “emergency” pharmaceutical medicines that are used as second-line drugs [46]. The recommendation of the American Epilepsy Society concludes that there was not strong support for the second-line treatment (level U) of phenytoin or levetiracetam; however, intravenous valproic acid administration has the same efficiency, which is also more tolerable than intravenous phenobarbital (level B) [11]. The NIH-sponsored Epileptic Treatment Test for Status Identified (ESETT) regards phenytoin, valproate, and levetiracetam equally, with strong evidence, for the treatment of convulsive SE in both children and adults [3].

Among pediatric emergency providers and neurologists, phenytoin or fosphenytoin remains the best-used antiseizure drug since the initial use of benzodiazepines [3]. This historical position, however, has been focused on little evidence that these drugs are better than other approaches, including levetiracetam, phenobarbital, or valproate. Latest meta-analysis approaches have shown that benzodiazepines have lower potency (50 percent) than phenobarbital (74 percent), valproate (76 percent), and levetiracetam (69 percent) [46].

Valproic acid is a commonly used antiseizure drug for modulating sodium and calcium channels, as well as GABA metabolism [47]. It is effective in both widespread and focal epilepsy, and it could be more effective for the treatment of epileptic illness in kids than in adults. Valproic acid has proved to be the most effective in comparison to traditional second-line antiseizure drugs [48].

Phenobarbital is a strong allosteric modulator for GABA-A receptors and is described as “emergent,” “urgent,” or “refractory” antiseizure drug under the guidance of the Neurocritical Care Society [18]. The standard phenobarbital IV dosage is 20 mg/kg and, if necessary, an extra 5–10 mg/kg. A new meta-analysis of benzodiazepine-refractory SE showed that 74% of the patients got phenobarbital effectiveness [46].

Levetiracetam is a further alternative for the treatment of SE [18]. The mechanism of action is not well known, but it should be remembered that it binds to a presynaptic glycoprotein and thereby reduces the release of a neurotransmitter [49]. Levetiracetam has a favorable pharmacokinetic profile and fewer interactions with few adverse reactions, so its use is appropriate for SE treatment. In some patients, seizure cessation has been achieved with levetiracetam at intravenous doses of 20–60 mg/kg, in small retrospective and observational trials, with the loading dose of 60 mg/kg as suggested by the American Epilepsy [11].

#### 6.2.2. RSE

The refractory epileptic status is distinguished by convulsions that persist irrespective of sufficient doses of initial treatment for antiseizure drugs. Refractory SE definition varies according to seizure durations (no time test, 30 min, 1 hour, 2 hours) or inability to respond to various antiseizure drugs. The guideline of the Neurocritical Treatment Society states that RSE is diagnosed when clinical or electrographic seizures continue following sufficient doses of initial benzodiazepine accompanied by an antiseizure drug of the second suitable type [18]. Despite its varied definitions, the ultimate goal is to provide prompt and concurrent management during RSE. In around 10%–40% of children with the epileptic condition, the RSE may occur [50]. A study of children with SE demonstrated that in 26%–45% of the patients, the duration of the status was over an hour, in 17%–25% of the patients lasted over a duration of two hours, and in 10% lasted over a period of four hours [51].

In several patients, the RSE lasts for several weeks or months despite the use of several drugs, and this condition is termed as malignant refractory status epilepticus or superrefractory status epilepticus [20]. De novo cryptogenic multifocal refractory SE, new-onset refractory status epilepticus (NORSE), and febrile epilepsy syndrome associated with inflammation (FIRES) are also often referred to as RSE [4]. Management of RSE has been historically studied in children [26]. While heterogeneity is indicated and management decisions are reported [52], some approaches either include additional medicines to antiseizure drugs like phenytoin/fosphenytoin, phenobarbital, valproate sodium, or levetiracetam or use intravenous or inhaled medicines to induce pharmacological coma. The Recommendation of the Neurocritical Treatment Society suggests administering untreated “urgent” control drug, followed by inducing pharmacological coma while the seizure remains or patient is directly subjected to pharmacological coma; these are acceptable choices for RSE management [18]. Even though the seizure appears to be fragmented, i.e., lesser frequency among subjects, continued antiseizure control measures (for example, administering phenytoin, valproate, levetiracetam, and phenobarbital) with pharmacologically induced coma are of utmost importance in mitigating any further relapses and fatal consequences [3, 53].

Few data are available on the use of midazolam, pentobarbital, and other anesthetics for status epilepticus [54]. An initial loading dosage of 0.2 mg/kg midazolam is administered followed by a 0.05–2 mg/kg/hour dose that is titrated to attain clinical or electrographic seizure suppression or EEG burst suppression as per the requirement. The initial loading of the pentobarbital dosage is typically 5–15 mg/kg (followed up by another 5–10 mg/kg if required) followed by a 0.5–5 mg/kg/hour injection, titrated if necessary for the suppression of the seizure. When convulsions persist after midazolam or pentobarbital treatment, it is important to supplement the treatment by administering additional drug doses to quickly stop the convulsions. Increasing the infusion rate without additional bolus dosing would result in a very slow serum rise, which is incompatible with the intention of rapidly stopping a seizure. Anesthetics, such as isoflurane, are often successful in burst suppression and ending seizures, but they can also induce hypotension. Propofol can quickly stop seizures and evoke burst suppression, but it can cause propofol infusion syndrome and, therefore, is seldom used in infants [3].

It remains unknown if the EEG-based treatment target is to stop seizures or cause burst suppression. Electrographic seizure termination of burst suppression can be an acceptable target in the guideline of the Neurocritical Care Society [18]. The period of the patient in a pharmacological coma is unknown. A recent study reported that pharmacological coma should last for 24 hours for all age groups [52]. The Neurocritical Care Society advises inducing a pharmacological coma for 24–48 hours before continuous infusion drugs are drawn down steadily [18].

#### 6.2.3. NCSE

The typical first and second lines of treatment employed in SE are not applicable in this pathological condition. Antiseizure medication and anesthetic drugs constitute the mainstay for its treatment [10]. Antiseizure drugs are IV-based topiramate, pregabalin, clobazam, and perampanel.

Anesthetic: commonly utilized anesthetic agents include midazolam, propofol, and barbiturates (pentobarbital in the USA and thiopental in Europe) [24].

### 6.3. Other Therapies Used in the Management of SE

#### 6.3.1. Recurrent Transcranial Magnetic Stimulation (rTMS)

As the name indicates, it is a repetitive therapy involving the direct application of magnetic stimulations to the patient's brain, facilitating transient electrical (activation or inhibition) activity in the underlying regions. rTMS is thus a recent promising, noninvasive, nonpharmacological intervention of 30–60 min duration performed in a non-ICU setting. Several case studies cited in the public domain have affirmed the good toleration of rTMS among epileptic patients with minimal side effects and complications [55, 56]. Low-frequency rTMS has shown significant seizure reduction, although scarcity in its documented safety and efficacy data currently limits its widespread use [10].

#### 6.3.2. Electroconvulsive Therapy (ECT)

ECT is markedly an efficient, safe, and established methodology, recommended in psychiatry practice guidelines in managing several psychiatric disorders, drug-resistant variants of SE. It involves brief electrical stimulation of the patient's brain in an anesthetized condition, under the mandatory surveillance of a neurologist and anesthesiologist. The exact mechanism of action remains unknown, although some studies have demonstrated the triggering of presynaptic GABA transmission pathways for seizure control. A meta-analysis of eight case studies has shown around 80% reduction in seizures with a complete recovery rate of 27% among patients, which in turn suggests that ECT is one of the viable alternatives for the management of RSE and other resistant variants of SE [10, 26].

#### 6.3.3. Immunomodulation

The use of the immunoglobulins is based upon the findings that antibodies directed against the voltage-gated potassium channels and the NMDA receptor may result in superrefractory GCSE [26]. Increasing evidence suggests that inflammation plays a significant role in epileptogenesis, especially the activation of specific inflammatory signaling pathways, such as the interleukin-1 receptor/ Toll-like receptor (IL-1 R/TLR) pathway [26].

Immunoglobulins, steroids, and plasma exchange (PLEX) are the key components in the immunological therapies that are reported in several case studies for the treatment of SRSE. Two case series were reviewed in adult patients who received intravenous immunoglobulin (IV IGs) for NORSE with no clear etiology [10]. In a study involving five patients, two of them received IV IGs along with high steroid dose, and one patient was given only high-dose steroid. No significant neurological deficit was observed in any of the three recovered patients. In another study, out of the seven patients who received immunotherapy treatment, three patients received IV IGs, five patients died, and two survived [10]. The data regarding the treatment of SRSE with PLEX are less as compared to steroids and immunoglobulins. In the two patients treated with PLEX for SRSE, one responded positively to the treatment, and in another, sedation was weaned off [10].

Immunomodulation therapies (i.e., corticosteroids, IV immunoglobulins, and plasmapheresis) have been administered in superrefractory GCSE cases [26]. Administration of steroids may also decrease intracranial pressure, reverse blood-brain barrier opening, and reverse GABAergic inhibition, which may influence persistent seizures [26].

#### 6.3.4. Hypothermia

Evidence-based findings of thermal hypothermia's role in mitigating different cerebral injuries are copious in both adults and pediatric subjects. Thermal hypothermia is one of the most researched nonpharmacological approaches exhibiting potential benefits in managing refractory and superrefractory SE. This targeted temperature therapy (31–35°C) for 1 to several days, in combination with other antiepileptic drugs, like ketamine, thiopental, and midazolam, has proven efficacy in controlling refractory SE [57]. In another study, patients gained control over seizures with therapeutic hypothermia at 31°C–35°C for 20 to 61 hours, using endovascular cooling [26, 58]. Kim et al. reported successful treatment of a 46-year-old person with hypothermia as a sole therapy [59]. However, Legriel et al. reported no significant benefits of hypothermia (32–34°C for 24 hours) against standard antiseizure therapy in critically ill patients with CSE [57]. Several adverse effects, like reduced renal clearance, cardiac arrhythmia, intravascular coagulation, etc., have been associated with this therapeutic thermal treatment [26].

#### 6.3.5. Ketogenic Diet (KD) and Modified Atkins Diet

A ketogenic diet or modified Atkins diet includes high fat, adequate protein, and low carbohydrate content. Recent reports show promising results of such diet therapies in controlling refractory SE in children and adults, which has also resulted in great research momentum in this domain. A 4:1 ratio of fat + protein to carbohydrate content has shown convincing improvement in 9 cases of superrefractory SE caused by febrile illness. Another study with 4 children and 1 adult with SE associated with viral infection demonstrated a 50% decrease in the frequency of seizures [26]. Thakur et al. (2014) described 90% efficacy in treating critically ill adults with superrefractory SE with a ketogenic diet [60]. Similar promising results have shown 50% reduction in seizure activity, in adults, when ketogenic diet and modified Atkins diet were introduced to the SE management [61]. Thus, it can be concluded such diet therapies offer promising adjunctive strategies in the treatment of SE.

#### 6.3.6. Vagus Nerve Stimulus (VNS)/Pacemaker of Brain

Another strategy of dealing with SE is by inducing electrical stimulation of the vagus nerve by surgically implanting a pacemaker-like device termed as vagus nerve stimulation or pacemaker of the brain [62]. An exact mechanistic model of VNS is not completely elucidated, but direct or indirect activation of vagal afferents through electrical stimulation by such device helps in refraining the episodes of seizures. Control of refractory SE in a 13-year-old subject with VNS resulted in immediate cessation of seizures [26]. VNS has been found to be most effective among pediatric patients, wherein the frequency of seizures reduced by around 50–65% cases with less than 5% remission rate [26].

## 7. Conclusion and Way Ahead

SE is a complex neurological disorder observed mostly in the two distinct age groups, children and older adults. It results in significant mortality and morbidity rates and is the second-largest neurological disorder resulting in death.

The present study discusses the diversified concept of SE concerning its definition, occurrence, types, etiology, diagnosis, and treatment. Significant animal and human data generated throughout the years has tremendously helped understand the underlying molecular mechanism leading to the pathogenesis of SE and its types. It has immensely benefitted in developing targeted diagnostic and therapeutic measures for SE. However, the paucity of both human as well animal data even today limits the fundamental understanding of SE variants.

Technological advances in synthetic biology, engineered cells or genes as a medicine, will help develop effective diagnostic as well as therapeutic measures in identifying and curbing refractory SE. Harmonized and validated guidelines based upon clinical trials need to be thoroughly researched. Furthermore, especially in developing countries, there is a need to make the antiseizure drugs with lower side effects more accessible and assist in cost-effective SE management.

## Figures and Tables

**Table 1:** Timeframe for SE as per the ILAE guidelines [14].

Sr. no.	Seizure type	T1	T2
(1)	Tonic-clonic	5 min	30 min
(2)	Focal SE with impaired consciousness	10 min	>60 min
(3)	Absence of SE	10–15 min	Unknown

**Table 2:** Classification of SE based on different parameters.

Sr. no.	Classification basis	Type	Reference
(1)	Origin of epileptic discharge	(1) Focal	[16]
		(2) Generalized	
		(3) Indeterminate or unclassifiable	

(2)	Etiology	(1) Acute symptomatic	[16, 17]
		(2) Remote symptomatic	
		(3) Remote symptomatic with an acute precipitant	
		(4) Progressive encephalopathy	
		(5) Febrile	
		(6) Idiopathic/cryptogenic	

(3)	Clinical	(1) Generalized convulsive SE (GCSE)	[1]
		(2) Secondary generalized SE	
		(3) Nonconvulsive SE	
		(4) Partial SE	
		(5) Neonatal SE	
		(6) Unclassified	

(4)	EEG correlates	(1) Location	[14]
		(2) Name of pattern	
		(3) Morphology	
		(4) Time	
		(5) Modulation	

(5)	Age	(1) Neonatal	[14]
		(2) Infancy	
		(3) Childhood	
		(4) Adolescence or adulthood	
		(5) Elderly	

(6)	Underlying cause	(1) Primary central nervous system disorders	[1]
		(2) Metabolic disorders	
		(3) Systemic disorders	
		(4) Noncompliance with drugs	

**Table 3:** Consolidated summary of SE and its types.

Sr. no.	Variant	Definition and key features	Causes	Diagnosis	Treatment	Reference
(1)	SE	Seizure that persists for a significant period of time and occurs frequently is termed SE Exhibits high morbidity and low mortality rates	Imbalance at cellular level pathways operating in CNS	EEG	Antiseizure drugs Anesthetic agents	[3, 10, 14]

(2)	RSE	SE which is incurable with two antiseizure drugs of which benzodiazepine is the one and the seizure lasts for around 1–2 hours Exhibits low mortality and high morbidity	Duration of SE CNS infections Massive stroke Brain tumors	Comprehensive metabolic profile Blood tests	Combination of antiepileptic drugs Electroconvulsive therapy Recurrent transcranial magnetic stimulation (rTMS)	[1, 3, 10, 22]

(3)	SRSE	SE that continues up to 24 hours or more even after the use of anesthetic therapy Exhibits high mortality and morbidity	Duration of SE CNS infections Metabolic disorders	Cerebrospinal fluid tests Blood tests	Immunomodulation therapies with immunoglobulins (IGs) and steroids Plasma exchange Epilepsy surgery	[10, 26]

(4)	NORSE	Occurs due to prolonged period of refractory seizures with no identifiable cause . Exhibits high mortality and morbidity	Autoimmune disorders Unidentified viral infections	Careful investigation of clinical history Continuous EEG monitoring Brain CT and MRI scan Cerebrospinal fluid tests Blood tests	Newer analogs of antiseizure drugs Anesthetic agents	[10, 24, 27]

(5)	FIRES	Subjects experience a nonspecific febrile illness followed by the onset of seizure activity lasting around 1–2 weeks. Exhibits high mortality and morbidity	Autoimmune paraneoplastic syndrome	Careful investigation of clinical history Prolonged EEG Lumbar puncture Blood tests Genetic tests MRI	Ketogenic diet Cannabidiol IGs (immunoglobulins) Monoclonal antibodies	[10, 24, 25, 27]

**Table 4:** Antiseizure drugs and their mechanism of action.

Sr. no.	Antiseizure drugs	Mechanism
(1)	Carbamazepine, eslicarbazepine acetate, felbamate, lamotrigine, oxcarbazepine, phenytoin, pregabalin, topiramate, valproate, zonisamide	Voltage-gated Na^+^ channel blocker
(2)	Gabapentin, pregabalin, ethosuximide, topiramate, valproate, zonisamide, lamotrigine	Voltage-gated Ca^2+^ channel blocker
(3)	Barbituates, benzodiazepines, felbamate, topiramate, propofol	GABA receptor antagonist
(4)	Vigabatrine, tiagabine	GABA antagonist
(5)	Perampanel	AMPA antagonist
(6)	Felbamate, ketamine, propofol, magnesium	NMDA receptor antagonist

## Data Availability

No data were used to support this study.
